# Lymphocyte proliferation to mycobacterial antigens is detectable across a spectrum of HIV-associated tuberculosis

**DOI:** 10.1186/1471-2334-9-21

**Published:** 2009-02-23

**Authors:** Timothy Lahey, Mecky Matee, Lillian Mtei, Muhammad Bakari, Kisali Pallangyo, C Fordham von Reyn

**Affiliations:** 1 Dartmouth Medical School, Lebanon, NH, USA; 2 Muhimbili University of Health and Allied Sciences, Dar es Salaam, Tanzania

## Abstract

**Background:**

Identifying novel TB diagnostics is a major public health priority. We explored the diagnostic characteristics of antimycobacterial lymphocyte proliferation assays (LPA) in HIV-infected subjects with latent or active TB.

**Methods:**

HIV-infected subjects with bacille Calmette Guérin (BCG) scars and CD4 counts ≥ 200 cells/mm^3 ^entering a TB booster vaccine trial in Tanzania had baseline in vivo and in vitro immune tests performed: tuberculin skin tests (TST), LPA and five day assays of interferon gamma (IFN-γ) release. Assay antigens were early secreted antigenic target 6 (ESAT-6), antigen 85 (Ag85), and *Mycobacterium tuberculosis *whole cell lysate (WCL). Subjects were screened for active TB at enrollment by history, exam, sputum smear and culture. We compared antimycobacterial immune responses between subjects with and without latent or active TB at enrollment.

**Results:**

Among 1885 subjects screened, 635 had latent TB and 13 had active TB. Subjects with latent TB were more likely than subjects without TB to have LPA responses to ESAT-6 (13.2% vs. 5.5%, P < 0.0001), Ag85 (18.7% vs. 3.1%, P < 0.0001), and WCL (45.7% vs. 17.1%, P < 0.0001). Subjects with active TB also were more likely than those without active TB to have detectable LPA responses to ESAT-6 (38.5% vs. 8.1%, P = 0.0001), Ag85 (46.2% vs. 8.5%, P < 0.0001), and WCL (61.5% vs. 27.0%, P = 0.0053). In subjects with a positive TST, LPA responses to ESAT-6, Ag85 and WCL were more common during active TB (p < 0.0001 for all tests). In diagnosing active TB, in vivo and in vitro tests of mycobacterial immune responses had sensitivity and specificity as follows: TST 84.6% and 65.5%, ESAT-6 LPA 38.5% and 92.0%, Ag85 LPA 46.2% and 91.5%, and WCL LPA 61.5% and 73.0%. Detectable LPA responses were more common in patients with higher CD4 counts, and higher HIV viral loads.

**Conclusion:**

Lymphoproliferative responses to mycobacteria are detectable during HIV-associated active TB, and are less sensitive but more specific than TST.

**Trial registration:**

ClinicalTrials.gov Identifier NCT00052195.

## Background

Tuberculosis (TB) is a major cause of death in HIV-infected people worldwide [1], yet, current approaches to TB diagnosis in HIV-infected people are inadequate [2]. Therefore, identifying novel TB diagnostics is a major international public health priority [3]. Interferon gamma (IFN-γ) release assays (IGRA) are under active investigation for the diagnosis of latent and active TB [4-7]. Since diminished IFN-γ release is a cardinal antimycobacterial immune defect in HIV-infected people [8-14], and vulnerability to active TB in HIV-infected people increases with increasing immunodeficiency [15], HIV-related immunodeficiency has the potential to compromise IGRA accuracy. Thus, it will be important to investigate additional methods of diagnosing latent and active TB.

Antimycobacterial lymphocyte proliferation is also detectable in HIV-infected subjects with latent and active TB [8,16-18], and therefore merits investigation for the diagnosis of latent and active TB in HIV infection. However, because antimycobacterial lymphocyte proliferation can also be impaired during HIV infection [8,16,17], it will be important to evaluate the relation between stage of HIV disease and LPA diagnostic utility. Accordingly, we evaluated LPA responses in subjects with and without latent or active TB in a cohort of HIV-infected BCG-immunized subjects with CD4 counts ≥ 200 cells/mm^3 ^entering a TB booster vaccine trial in Tanzania [19,20]. This is the first report to characterize the diagnostic characteristics of LPA during active TB.

## Methods

### Study subjects and data collection

The Dar Dar Study was a phase III randomized placebo-controlled trial of a prime-boost vaccine strategy for the prevention of HIV-associated tuberculosis in adults in Dar es Salaam, Tanzania [20]. Enrollment for this study occurred from 2001 to 2005, and study follow up continued through January 2008. All subjects were screened with a standardized interview, including history of prior treatment for active tuberculosis, physical examination, CD4 count and single view chest x-ray. Eligible subjects who gave informed consent were enrolled if they have two positive ELISA antibody tests for HIV, a CD4 count ≥ 200/mm^3^, a BCG scar and no evidence of active tuberculosis (TB). At enrollment, subjects were further evaluated with three expectorated sputum samples collected for AFB smear and culture, and a single mycobacterial blood culture. Per protocol, subjects with active TB detected immediately at baseline were ineligible for enrollment and thus discharged from the study with immunological studies not performed. In contrast, subjects with baseline active TB that was not detected immediately, but later identified via baseline mycobacterial cultures of sputum or blood, were not yet discharged from the study and as a result had immunological tests performed. Subjects in this latter category, with delayed recognition of baseline TB on whom immunological studies were performed, are the subject of this report. Human experimentation guidelines of the United States Department of Health and Human Services, as well as that of the Committee for the Protection of Human Subjects at Dartmouth College and the Research Ethics Committee of the Muhimbili University of Health and Allied Sciences, were followed in the conduct of this research. This study is registered through the National Institutes of Health (NCT00052195).

### Definitions of TB

Subjects with a reactive tuberculin skin test (TST) at ≥ 5 mm but no active TB were deemed to have latent TB. Subjects were designated as having active TB if they met a study definition of definite or probable TB as determined by the consensus of a three person expert panel (Table 1).

**Table 1:** Case definitions.

Definite TB	1. One or more sputum cultures positive for *Mycobacterium tuberculosis *(MTB) with ≥ 10 colony forming units (CFU); or,
	2. Two or more sputum cultures with 1–9 CFU of MTB (indeterminate MTB culture); or,
	3. Two or more positive sputum smears for acid fast bacilli (AFB); or,
	4. One or more cultures for MTB from the blood or other sterile body site.
Probable TB	1. Positive chest x-ray plus either
	a. one positive sputum AFB smear, or,
	b. one indeterminate MTB culture result; or,
	2. Clinical symptoms/signs plus either
	a. one positive sputum AFB smear, or,
	b. one indeterminate MTB culture result; or,
	3. Clinical symptoms/signs and a positive x-ray plus a response to anti-TB therapy; or,
	4. One positive sputum AFB smear from a sterile site plus clinical symptoms/signs of tuberculosis; or,
	5. Caseous necrosis on a tissue biopsy.

Latent TB	1. Tuberculin skin test ≥ 5 mm
	2. No definite or probable TB.

AFB, acid fast bacillus; CFU, colony forming units; MTB, *Mycobacterium tuberculosis*; TB, tuberculosis

### Tuberculin skin testing

All subjects underwent TST with 0.1 mL tuberculin (RT-23, State Serum Institute, Copenhagen) as well as with 0.1 mL *Mycobacterium avium *sensitin (MAS, State Serum Institute, Copenhagen). The size of skin induration at the site at 48–72 hours was measured, and reactions ≥ 5 mm were considered positive.

### Lymphocyte proliferation assays (LPA)

LPA were conducted on freshly isolated and ficolled peripheral blood mononuclear cells using a standard five day ^3^H-thymidine incorporation method with media alone, 1 mcg/ml *M. tuberculosis *ESAT-6, 0.5 mcg/ml *M. tuberculosis *Ag85, or 0.5 mcg/ml *M. tuberculosis *whole cell lysate (WCL), with all antigens acquired through NIH, NIAID Contract No. HHSN266200400091C, entitled "Tuberculosis Vaccine Testing and Research Materials," awarded to Colorado State University. After incubation, 20 μl of 50 μCi/ml ^3^H-thymidine was added to wells for 24 hours, after which the cells were harvested onto filter paper and sent to the National Public Health Institute in Helsinki, Finland, for data acquisition on a scintillation counter. Results were expressed as a proliferation index (PI; counts per minute [CPM] of antigen stimulated cells divided by CPM of unstimulated cells), with a proliferation index of ≥ 5 considered positive. LPA results are shown for all assays, but equivalent results were derived when identical analyses were undertaken after excluding assays if the positive control (phytohemagglutinin [PHA] 2.5 μg/ml) PI was < 3, or less than the unstimulated condition, or if the CPM for the unstimulated cells was over 5000.

### Supernatant IFN-γ levels

IFN-γ levels in frozen cell culture supernatants were evaluated after 5-day stimulation with the same antigens using a standard enzyme linked immunosorbent assay (ELISA; R&D Systems). Results were considered positive if IFN-γ level was greater than or equal to two standard deviations above the negative mean.

### Statistical analysis

Standard descriptive statistical methods were used to characterize baseline demographical characteristics of the study population. Comparisons between demographic characteristics and immune responses between subjects with no TB, latent TB and active TB were made using analysis of variance (ANOVA). Comparisons between two groups were made using t tests. P values less than 0.05 were considered statistically significant. The diagnostic utility of immune responses in TB was assessed using receiver-operating characteristic (ROC) analysis. Agreement between tests was assessed using Spearman's rank correlation coefficients. Trends in the relation between the risk of TB and the number of antimycobacterial immune responses were evaluated using the score test for trend of odds. Parameters demonstrating differences between TB and no TB groups in univariate analyses were incorporated into a logistic regression model. Correlations between the results of *in vivo *and *in vitro *immune assays, and with clinical parameters, utilized the Pearson chi-squared test. Data were analyzed on STATA 9 (College Station, TX) and Graph Pad Prism 4 (San Diego, CA).

## Results

The characteristics of the study subjects are shown in Table 2, including the 13 with delayed recognition of active TB at baseline. All subjects were HIV positive, had BCG scars and CD4 counts ≥ 200/mm^3^. Evaluable LPA results were available in 1855 subjects: IFN-γ results in 1542 subjects and TST results in 1815 subjects. Thirteen subjects had positive sputum cultures for MTB; eleven fit study criteria for definite TB and two for probable TB. Subjects with active TB had higher mean HIV viral loads (87,292.6 vs. 34023.0 copies/mL, P = 0.0066), and were more likely to have a positive TST (84.6% vs. 34.5%, P = 0.0002) when compared to all subjects without active TB.

**Table 2:** Characteristics of study subjects.

	Subjects without TB (n = 1206)	Subjects with latent TB (n = 635)	Subjects with active TB (n = 13)	P-value by ANOVA
Age, mean	33.0	33.5	34.1	0.5102

Male, % (n)	247 (20.5%)	193 (30.4%)	3 (23.1%)	< 0.0001

CD4 cells/mm^3^, mean	455	502	376	0.0001

HIV viral load, copies/mL, mean	37,286	28,365	87,293	0.0063

Previous TB, % (n)	98 (8.1%)	61 (9.6%)	2 (15.4%)	0.3865

On antiretrovirals, % (n)	44 (3.6%)	13 (2.0%)	0 (0%)	0.1366

TST, mm, mean	0.2	15.5	15.0	< 0.0001

TST ≥ 5 mm, % (n)	0 (0%)	635 (100%)	11 (84.6%)	< 0.0001

MAS, mm, mean	0.3	9.6	10.1	< 0.0001

MAS ≥ 5 mm, % (n)	835 (69.2%)	582 (91.7%)	10 (76.9%)	< 0.0001

ANOVA = analysis of variance; HIV = human immunodeficiency virus; MAS = *Mycobacterium avium *sensitin skin test; TB = tuberculosis; TST = tuberculin skin test

### LPA results during latent and active TB

**Figure 1:**
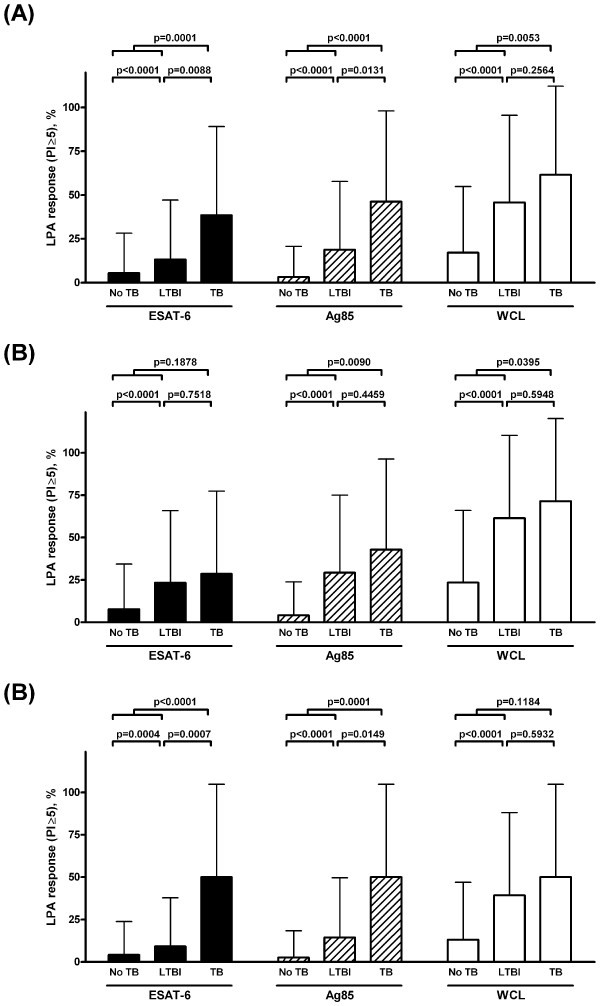
**Lymphocyte proliferation to mycobacterial antigens in subjects with a spectrum of tuberculosis**. The percentage of subjects with lymphocyte proliferation index of ≥ 5 to the indicated antigens is compared in subjects with active TB, latent TB and no TB in (A) all subjects, (B) subjects with a CD4 count < 350 cells/ul, and (C) subjects with a CD4 count ≥ 350 cells/ul. The proliferation index is the ratio of counts per minute after stimulation with indicated antigen over counts per minute in unstimulated condition. (Ag85, antigen 85; ESAT-6, early secretory antigenic target 6; LTBI, latent tuberculosis infection; TB, tuberculosis; WCL, *Mycobacterium tuberculosis *whole cell lysate)

Figure 1A and Table 3 show the LPA responses to mycobacterial antigens in subjects with no TB, latent TB and with active TB. Compared to subjects without TB, detectable LPA responses to ESAT-6, Ag85 and WCL were significantly more common in subjects with latent and active TB. A pattern of increasing LPA responses to all three antigens was seen when moving from no TB to latent TB to active TB.

**Table 3:** Prevalence of detectable lymphocyte proliferation to mycobacterial antigens in HIV-infected BCG-vaccinated adults with and without active TB.

Antigen	Subjects without TB (n = 13)	Subjects with latent TB (n = 635)	Subjects with active TB n = 1206)	P-value by ANOVA
PHA, % (n)	543 (45.0%)	278 (43.8%)	8 (61.5%)	0.4175

MVS, % (n)	26 (2.2%)	21 (3.3%)	0 (0%)	0.2756

ESAT-6, % (n)	66 (5.5%)	84 (13.2%)	5 (38.5%)	< 0.0001

Ag85, % (n)	38 (3.1%)	119 (18.7%)	6 (46.2%)	< 0.0001

WCL, % (n)	207 (17.1%)	290 (45.7%)	8 (61.5%)	< 0.0001

Ag85, antigen 85; ESAT-6, early secretory antigenic target 6; MVS, *Mycobacterium vaccae *sonicate; PHA, phytohemagglutinin; TB, tuberculosis; WCL, *Mycobacterium tuberculosis *whole cell lysate

### Agreement of LPA results

There was a significant correlation between LPA responses to different mycobacterial antigens: ESAT-6 vs. Ag85, Spearman's rho 0.4242, P < 0.0001; ESAT-6 vs. WCL, Spearman's rho 0.3646, P < 0.0001; Ag85 vs. WCL, Spearman's rho 0.4033, P < 0.0001.

### LPA responses by TST status during active TB

We compared LPA responses in subjects with active TB to LPA responses in all other subjects. In subjects with a positive TST, TB was more likely in subjects with detectable LPA responses to ESAT-6 (P < 0.0001), Ag85 (P < 0.0001) and WCL (P < 0.0001). The specificity of LPA to ESAT-6 and Ag85 was over 80% in subjects with a positive TST (Table 4).

**Table 4:** Operating characteristics of TST and LPA assays for the diagnosis of active TB.

	All subjects					*Subjects with TST ≥ 5 mm*				
	Sensitivity, %	Specificity, %	AUROC	PPV	NPV	Sensitivity, %	Specificity, %	AUROC	PPV	*NPV*

TST	84.6	65.5	0.7507	1.7	99.8	--	--	--	--	--

ESAT-6	38.5	92.0	0.6516	3.2	99.5	45.5	86.8	0.6611	5.6	*98.9*

Ag85	46.2	91.5	0.6882	3.68	99.6	54.6	81.3	0.6790	4.8	*99*

WCL	61.5	73.0	0.6728	1.58	99.6	72.7	54.3	0.6353	2.68	99.1

Ag85, antigen 85; AUROC, area under the receiver-operating characteristic curve, calculated as sensitivity plotted against 1-specificity; ESAT-6, early secretory antigenic target 6; LPA, lymphocyte proliferation assay; NPV, negative predictive value; PPV, positive predictive value; TB, tuberculosis; TST, tuberculin skin test; WCL, *Mycobacterium tuberculosis *whole cell lysate

### LPA results according to TST status in subjects without active TB

In the group of patients without active TB at baseline, detectable LPA responses were more common in subjects with a positive TST: ESAT-6 13.2% vs. 5.5%, P < 0.0001; Ag85 18.7% vs. 3.1%, P < 0.0001; and, WCL 45.7% vs. 17.1%, P < 0.0001.

### LPA operating characteristics

The sensitivity and specificity of TST for detecting TB were 84.6% and 65.5%, respectively. As shown in Table 4 the sensitivity of LPA assays was lower than TST in every case, but antimycobacterial LPA assays were generally more specific. The area under the ROC curve (AUROC) for TST was higher than for LPA at either PI cutoff. The positive predictive values for LPA responses to all antigens were low, as for TST. The negative predictive values for all LPA assays were approximately 99% regardless of TST status. Assay operating characteristics were not improved when combining multiple antigen responses (data not shown).

### LPA responses and CD4 count or HIV viral load

There was a weak but statistically significant negative correlation between CD4 count and the likelihood of LPA responses to ESAT-6 (Pearson r -0.1044, P < 0.0001), Ag85 (Pearson r -0.0744, P = 0.0013) and WCL (Pearson r -0.1162, P < 0.0001). In subjects stratified by CD4 count, LPA responses showed the same increasing pattern from no TB to latent TB to active TB, but in the low CD4 count group LPA was generally less able to discriminate between latent and active TB (Figures 1B-1C). No LPA response could discriminate subjects with latent TB from active TB in the CD4 < 350 stratum. The HIV viral load correlated weakly but positively with the likelihood of detectable LPA responses to ESAT-6 (Pearson r 0.1577, P < 0.0001), Ag85 (Pearson r 0.1277, P = 0.0008), and WCL (Pearson r 0.1131, P = 0.0029). Because of the relation of LPA responses to CD4 count and HIV viral load, we stratified our analysis of the correlation between active TB and the antimycobacterial immune response by CD4 count and, separately, HIV viral load (Table 5), finding that detectable mycobacterial LPA responses were associated with higher odds of TB at higher CD4 counts and higher HIV viral loads.

**Table 5:** Odds of active TB according to lymphocyte proliferation assays, stratified by CD4 count and HIV viral load.

Odds of TB if PI ≥ 5 to Antigen								
	By CD4 count strata			By HIV Viral Load Quartile				

Antigen	CD4 count ≥ 350*	CD4 count < 350	P value by Mantel-Haenszel method	Lowest	Second	Third	Highest	P value by Mantel-Haenszel method

ESAT-6	15.8	2.9	0.0003	0	0	12.2	6.8	0.0030

Ag85	15.2	5.9	< 0.0001	0	0	11.2	7.1	0.0022

WCL	3.3	4.8	0.0098	0	0	0	2.7	0.0333

* Of subjects with active TB, six had CD4 counts ≥ 350

Ag85, antigen 85; ESAT-6, early secretory antigenic target 6; PI, proliferative index; TB, tuberculosis; WCL, *Mycobacterium tuberculosis *whole cell lysate

### Multiple LPA responses related to greater TB risk

**Figure 2:**
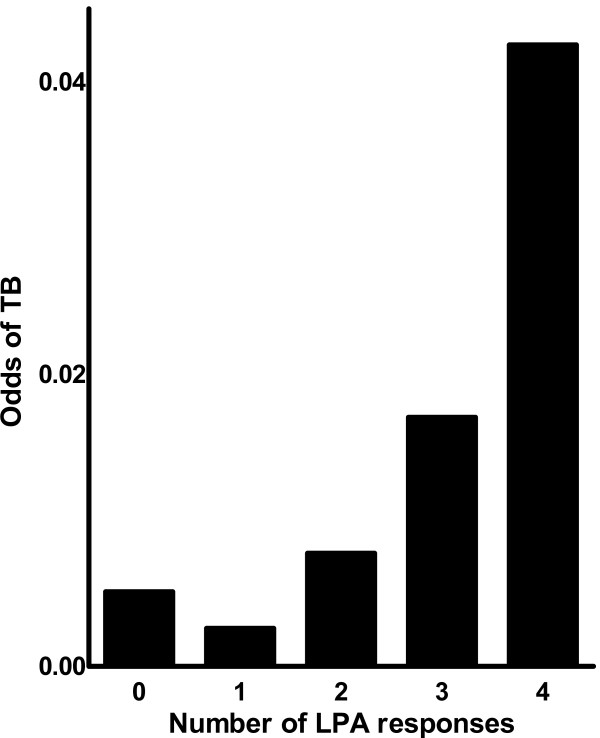
**Odds of active TB according to number of detectable antimycobacterial lymphoproliferative responses**. (LPA, lymphocyte proliferation assays; TB, tuberculosis)

Figure 2 shows the relation between the number of antimycobacterial responses by LPA and the odds of TB. An increasing number of antimycobacterial LPA responses were associated with increasing odds of TB (P = 0.0097).

### Multivariate model

In a multivariate logistic regression analysis adjusting for factors that differed between subjects with and without active TB, active TB was associated with LPA responses to ESAT-6 (OR 3.8, P = 0.036) and Ag85 (OR 3.2, P = 0.0760).

### IFN-γ results during latent and active TB

IFN-γ responses were more common in subjects with latent TB compared to those without TB (ESAT-6, 49.3% vs. 26.6%, P < 0.0001; Ag85, 38.6% vs. 24.8%, P < 0.0001; WCL, 78.4% vs. 42.8%, P < 0.0001), but IFN-γ responses were not consistently different between subjects with and without active TB (ESAT-6, 38.5% vs. 26.6%, P = 0.3366; Ag85, 23.1% vs. 24.8%, P = 0.8881; WCL, 76.9% vs. 42.8%, P = 0.0133). There was, however, a clear differentiation between IFN-γ responses to the positive control condition (PHA) compared to medium in all subjects (83.2% vs. 18.6%, P < 0.0001), in subjects without TB (85.0% vs. 19.6%, P < 0.0001), in subjects with latent TB (79.7% vs. 16.9%, P < 0.0001), and in subjects with active TB (69.2% vs. 15.4%, P = 0.0028). The absolute CD4 count correlated with the likelihood of having demonstrable IFN-γ responses to each mycobacterial antigen: ESAT-6, Pearson r = 0.1076, P < 0.0001; Ag85 Pearson r = 0.0488, P < 0.0356; and WCL Pearson r = -0.0868, P = 0.0002. By contrast, the HIV viral load weakly impacted only the likelihood of positive responses to ESAT-6, Pearson r = 0.0801, P = 0.0354.

## Discussion

An international effort is underway to find novel means of diagnosing and predicting TB in people with HIV [3]. We have shown that LPA responses to multiple mycobacterial antigens are enhanced during latent and active TB in HIV-infected subjects with CD4 counts ≥ 200 cells/mm^3 ^and prior BCG immunization, and that these responses are less sensitive but more specific than TST in diagnosing active TB.

No previous studies have examined the diagnostic utility of LPA responses during active TB in HIV-infected adults. One group showed that LPA responses to mycobacteria were detectable in HIV-infected adults with TB, and noted a decline in response frequency at lower CD4 counts and in comparison to subjects without HIV [8]. However, only 67% of patients in that study were BCG immunized, LPA responses were not characterized according to BCG immunization or TST status, and the diagnostic operating characteristics of the test were not explored. In a preliminary report on seven of the subjects with HIV and active TB described in the present study we showed that LPA responses to mycobacterial antigens were detectable but did not determine the operating characteristics of the LPA nor the relation of these results to CD4 counts or HIV viral load [18]. Others have detailed LPA responses to PPD and mycobacterial cell cultures in HIV-infected adults with latent TB [16,17], but these studies lack a description of the impact of HIV-related immunodeficiency or HIV viral load on assay results. Taken together, our data newly show that LPA responses to mycobacterial antigens are capable of detecting a spectrum of TB disease from latent to active and disseminated disease.

The ongoing search for novel TB diagnostic approaches in HIV-infected individuals results from the limitations of the TST in HIV-infected individuals, including decreased sensitivity at low CD4 counts and the potential for false positive results after BCG vaccination [21-23]. Here, we have shown that LPA is less sensitive than TST, but more specific. Importantly, we did not examine the comparative sensitivity of LPA and TST at CD4 counts under 200 cells/ul, since all subjects in this study had CD4 counts over 200 cells/ul. Since other immunological assays for the detection of TB have shown increased sensitivity when responses against multiple antigens were combined [4,24], this approach has the potential to increase LPA sensitivity. The higher specificity of LPA likely results from the use of more TB-specific antigens ESAT-6 and Ag85, and is particularly important in BCG-immunized subjects such as are prevalent in areas of high TB endemicity. Even in subjects with a positive TST, subjects lacking LPA responses to mycobacterial antigens were highly unlikely to have active TB.

IGRA's are the current frontrunners in the race to replace TST [4,5,25]. The sensitivity of detectable LPA responses to mycobacterial antigens reported here is lower than that reported previously for IGRA assays during active TB. This may be due to different test characteristics of IGRA vs LPA, or the fact that commercial IGRA assays employ a mycobacterial antigen (CFP-10) that we did not use in the present LPA assay. IGRA sensitivity in HIV-infected subjects who recently completed therapy for TB was 71–81%, whereas during culture positive TB in HIV-infected subjects the sensitivity of IGRA was 85% [26,27]. In both studies, BCG vaccination was not universal, although in both papers the authors reported that BCG immunization had no impact on IGRA sensitivity.

Improving the sensitivity of LPA during active HIV-associated TB nonetheless will be critical, as the sensitivity values we observed are inadequate for clinical use. However, LPA sensitivity may be increased by using alternate TB antigens in combination, such as was done with IGRA [24]. Furthermore, we suspect the estimates of LPA sensitivity in our study are conservative, since the subjects with active TB reported here were diagnosed late after being scrutinized for clinical trial enrollment, and thus may have more clinically and immunologically subtle TB disease than would be identified during routine clinical care.

The specificity of LPA assays in the present study is similar to that of IGRA. IGRA specificity has been reported to be 98.6% during active TB in HIV-negative subjects in Japan, [5], but in areas with high TB prevalence rates this figure is expected to be lower [28]. Accordingly, in BCG-immunized and HIV positive adults without active TB in Zambia, the specificity of IGRA was 71% for responses to ESAT-6 alone and 57% to ESAT-6 and CPT-10 combined [24]. These specificity values are lower than those we report for LPA, although no head-to-head comparison has yet been done. Since the TB epidemic flourishes largely in high prevalence areas where BCG vaccination is common, and in people with HIV co-infection, we believe it will be important to evaluate alternate platforms for diagnosing TB or TB risk in HIV-infected subjects from high TB prevalence areas [25].

Our data show that LPA responses are more predictive of TB risk in subjects with higher CD4 counts [16,17]. Importantly, all subjects in this study had CD4 counts above 200 cells/mm^3^, so it will be important to evaluate the diagnostic utility of LPA in subjects with more profound HIV-related immunodeficiency. This is particularly important since IGRA based on the enzyme-linked immunospot (ELISPOT) technique appear not to lose sensitivity at lower CD4 counts [29,30] although the impact of progressive HIV-related immunodeficiency on IGRA accuracy is still under study. While in prior studies antiretroviral therapy increased the likelihood of detectable LPA responses, patients in our cohort with higher HIV viral loads were more likely to have detectable lymphoproliferative responses to mycobacteria. Since antiretroviral therapy was rare in our cohort, the viral loads reported here reflect untreated values, and thus we posit that the relation between the HIV viral load and TB risk is different in untreated vs. treated subjects. It is possible that a high HIV viral load off therapy may contribute to greater HIV-related immunological dysfunction and thus vulnerability to TB disease, whereas treated patients with a lower viral load on therapy may be more likely to have detectable responses via antimycobacterial immune reconstitution. It is also possible that the higher viral loads were a consequence of new active TB [31].

HIV and TB intersect most lethally in the developing world. Thus, it is important to consider whether conducting the LPA – a complicated five day assay involving low-level radioactivity – is feasible in the developing world. We generated these data in Tanzania, but the assessment of lymphocyte radioactivity (which is proportionate to proliferation in this assay) was carried out in Finland. Further, all assays were conducted through a US-funded clinical trial with an intensive focus on quality control. Accordingly, we suspect that only larger medical centers in the developing world would have the capacity to conduct LPA assays.

Despite an extensive literature that suggests that IFN-γ responses to mycobacterial antigens are decreased but detectable in HIV-infected individuals [8,10,14,26,27], we did not see differences in IFN-γ responses in subjects with and without TB. One reason for this discrepancy may be differential assay duration. While the IFN-γ results here stem from five day incubation assays, currently commercially available assays employ one day of incubation. One day assays measure effector cell IFN-γ release, while five day assays likely measure IFN-γ release from effector and central memory T cells [32,33]. As a result, our five day IFN-γ assays may fail to discriminate between subjects with novel effector T cell responses generated during active TB and those with pre-existing memory T cell responses to TB. Despite careful attention to quality control in this developing world setting, a small proportion of IFN-γ assays lacked PHA responses or had detectable responses to the medium control. Assay failure thus has the potential to contribute to our IFN-γ assay's inability to detect differences between subjects with active TB or no TB, although notably the assay did discriminate between subjects with latent TB and no TB. Another potential reason for the discrepant IFN-γ results in this study is that certain mycobacterial antigens may be more efficient at generating IFN-γ responses than others. Commercially available assays of IFN-γ target ESAT-6 as well as CFP-10 with and without TB7.7 [4,25], and others have demonstrated detectable IFN-γ responses in HIV-infected subjects with active TB using purified protein derivative (PPD) [8,14] Our assay, which assessed responses to ESAT-6, Ag85 and WCL, may be less likely to generate IFN-γ responses that distinguish between subjects with and without TB. At a minimum, our data suggest that incubation period and antigen choice are critical variables in IFN-γ assays for TB.

Our data show that detectable LPA responses to TB are associated with TB infection, but not necessarily with effective cellular immune protection against disease. This is consistent with the known association between TST results and TB risk [34,35]. This model has important implications regarding the conduct of TB vaccine trials: it will be vitally important to determine not only whether TB vaccines generate antimycobacterial immune responses, but whether these immune responses correlate with disease protection.

## Conclusion

In conclusion, an assay of antimycobacterial lymphocyte proliferation was more specific but less sensitive than TST in detecting active TB in HIV-infected and BCG-immunized patients from a high TB prevalence area. Given the potential for improving LPA sensitivity by using a different panel of mycobacterial antigens and assessing it in subjects with more clinically overt active TB, LPA merits continued evaluation as a novel diagnostic tool for HIV-associated TB.

## Competing interests

The authors declare that they have no competing interests.

## Authors' contributions

TL helped design the study, performed data analysis and drafted the manuscript; MM helped with data acquisition and manuscript preparation; LM helped with data acquisition and manuscript preparation; MB helped with data acquisition and manuscript preparation; KP helped with data acquisition and manuscript preparation; CFR conceived the study, participated in its design and conduct, and helped to draft the manuscript. All authors read and approved the final manuscript.

## About the authors

The authors of this manuscript participate in the Dar Dar Health Study, a randomized placebo controlled trial of whole inactivated *Mycobacterium vaccae*, a novel TB booster vaccine given to 2,000 HIV-infected adults in Dar es Salaam, Tanzania.

## Pre-publication history

The pre-publication history for this paper can be accessed here:

http://www.biomedcentral.com/1471-2334/9/21/prepub
